# Drug shortage in South Korea: machine learning-based prediction models and analysis of duration and causal factors

**DOI:** 10.3389/fphar.2025.1608843

**Published:** 2026-01-09

**Authors:** Hyun Soo Roe, Da Eun Ko, Seo Yun Nam, Jae Eun Oh, Su Min Park, Se Hee Lee, Jong Hyuk Lee

**Affiliations:** 1 College of Pharmacy, Chung-Ang University, Seoul, Republic of Korea; 2 College of Pharmacy, Sookmyung Women’s University, Seoul, Republic of Korea

**Keywords:** drug shortage, feature importance, public health, random Forest model, shortage cause, shortage duration, supply chain

## Abstract

**Background:**

Drug shortages remain a critical challenge for healthcare systems in South Korea. This study aimed to develop predictive models to forecast drug shortage duration and identify their underlying causes.

**Methods:**

Using 1,054 regulatory-reported drug shortage cases from 2018 to 2024 obtained from the Korean Ministry of Food and Drug Safety (KMFDS), we developed two machine learning models: (1) Model 1 to estimate shortage duration ranges, and (2) Model 2 to classify shortage causes into seven categories. Eighteen features related to drug shortages were included based on relevance and data availability. Key predictors were identified using Random Forest feature importance.

**Results:**

Model 1 achieved an accuracy of 62%, with Shortage Incidence Frequency being the most influential variable (importance = 0.152). In Model 2, weighted precision, recall, and F1-score all exceeded 70%, indicating robust performance despite imbalanced class distributions. The most important predictors for cause classification included Shortage Incidence Frequency, Existence of alternative drugs with the same ingredient, and Business size of the Marketing Authorization Holder.

**Conclusion:**

The KMFDS should continuously monitor drugs with repeated shortage episodes through regular reporting, early-warning systems, and supply-risk assessments. Incorporating supply-side indicators—particularly those related to economic feasibility—into national surveillance programs may help prevent shortages and mitigate their duration. By identifying key predictors associated with shortage causes, this study provides evidence to guide policy prioritization and targeted interventions.

## 1 Introduction

The term “drug shortage” lacks a globally standardized definition, with significant variations or no definitions at all across countries (World Health Organization, 2015). From a supply perspective, drug shortages refer to the inadequate availability of medicines, health-related products, and vaccines required to meet public health and patient needs within the healthcare system. On the demand side, shortages arise when demand exceeds supply at any point in the supply chain, potentially leading to stockouts and an inability to meet clinical needs if not addressed promptly (Acosta et al., 2019; Health Canada, 2024).

Drug shortages impose significant societal costs on the healthcare sector, including reduced patient access to medicines, increased healthcare expenses, disruptions in the pharmaceutical markets, heightened anxiety among healthcare providers, and delays in developing innovative drugs (Fox et al., 2014; Pauwels et al., 2015; Shukar et al., 2021). During the COVID-19 pandemic, the global value chain for biopharmaceuticals faced unprecedented threats, intensifying global concerns about drug shortages. One study reported findings from a five-year study that emphasized the growing challenge of drug shortages and pointed the importance of proactive strategies, including investing in technology, strengthening supplier relationships, and advocating for policy reforms (Ayati et al., 2020; Sallam et al., 2024). Addressing this challenge is a critical issue for national healthcare systems (Ivanov and Das, 2020; Ayati et al., 2020; Piatek et al., 2020).

The prediction of drug shortages, time to shortage, and recovery period following a shortage, depending on the characteristics of the drug, are key factors in managing drug shortages (Tucker and Daskin, 2021). In Canada, a study was conducted to develop a machine-learning model to predict drug shortages using data from 22 pharmacy sales and historical drug shortage records (Pall et al., 2023). The model was able to predict shortage classes (none, low, medium, and high) with 69% accuracy 1 month in advance. Despite the lack of inventory data from drug manufacturers and suppliers, this demonstrates meaningful performance given limited data.

In South Korea, the pharmaceutical industry predominantly produces generic drugs and relies heavily on imported active pharmaceutical ingredients (APIs) as well as new drugs, making the supply chain sensitive and vulnerable to shortages. To address these challenges, in 2019, the government developed a government-led drug supply disruption prediction model in collaboration with the Korea Orphan and Essential Drug Center, using data from reports on drug supply interruptions and shortages to address drug shortages (National Institute of Food and Drug Safety Evaluation, 2019). In addition, to proactively identify supply and demand imbalances, they implemented a supply and demand forecasting project utilizing artificial intelligence. To address these imbalances, they expanded the list of nationally essential drugs and reinforced support for production (Seoul Pharmaceutical Association, 2023). However, the limitations of this model-designed only to provide real-time alerts for potential shortages-prevented it from addressing the underlying problems, leading to a significant increase in the number of drugs with unstable supply despite the expansion of national essential drugs (National Institute of Food and Drug Safety Evaluation, 2019; Seoul Pharmaceutical Association, 2023).

Thus, a retrospective analysis of 1,054 regulatory-reported drug shortages predictive system is urgently required to effectively address drug shortages. While the previous model aimed to provide real-time alerts for potential shortages, we analyze drugs that have already undergone shortages, examining their shortage duration range and cause to identify key features relevant for prediction. Also, unlike the previous model, our dataset includes information not only on the causes of shortage but also on their duration ranges. Therefore, this study aims to develop two types of models: one for predicting the causes of drug shortages and another for estimating their duration range. Identifying key features with high importance in each model could help to determine the factors crucial for predicting the occurrence of shortages based on their causes and estimating their duration in South Korea. By providing not only early indications of whether a shortage may occur but also insights into why it happens and how long it is likely to persist, our models allow decision-makers to implement more strategic and timely interventions. These actionable insights support more targeted policy responses and early risk mitigation, ultimately contributing to a more resilient drug supply system in South Korea.

## 2 Methods

### 2.1 Data source

Data on 1,054 cases of drug shortages reported by pharmaceutical companies to the Korean Ministry of Food and Drug Safety (KMFDS) between 2018 and 2024 were collected from the Drug Safety System on the KMFDS website (https://www.mfds.go.kr). All 1054 cases reported during this period were included in the analysis.

### 2.2 Features

Eighteen variables of Model 1 and 2 related to drug shortages were selected (Supplementary Table S1). These variables were chosen based on drug shortage factors (referred to as “features”) identified in Canada, the United States, and South Korea, as well as variables selected from the drug supply disruption prediction model in South Korea (Pall et al., 2023; National Institute of Food and Drug Safety Evaluation, 2019; United States Government Accountability Office, 2016; U.S. Food and Drug Administration FDA, 2020).

Features were selected based on the following criteria: (1) data available from open sources, such as the Drug Safety system in the KMFD, and (2) continuous or nominal variables with convertible numerical values. For the Year of approval, the corresponding decade was considered more meaningful than the specific year. Thus, data were categorized into three intervals (1960–1970s, 1980–1990s, and after the 2000s) to ensure an even distribution (Shukar et al., 2021; Pall et al., 2023; Health Insurance Review and Assessment Service, 2023; Liu et al., 2021). Decade-based grouping was used for data balance, but we acknowledge that this approach may obscure more detailed temporal variation. Drug shortage causes were systematically classified into seven major categories in this study as shown in Supplementary Table S2.

## 3 Modeling

### 3.1 Data processing

The raw dataset contained 1,054 records reported between 2018 and 2024. Ambiguous entries such as “?” or “–” and remaining missing values were recoded as 0. For Model 1, shortages with missing duration (Class 0, 0.1%) were removed, leaving 1,053 records. The remaining five duration classes were used as ordered target categories: Class 0 (missing duration), Class 1 (<1 month), Class 2 (1–6 months), Class 3 (6–12 months), Class 4 (≥12 months), and Class 5 (permanent discontinuation/no recovery). For Model 2, all 1,054 records were retained, and each shortage cause was modeled as a separate binary one-vs-rest classifier. The original multi-category “Cause of shortage” variable was therefore converted into six binary targets. All features were converted to numeric format, and the route of administration variable was one-hot encoded using get_dummies, as required for non-ordinal categorical inputs in Random Forest models. No normalization was applied to continuous variables, as tree-based models such as Random Forest are inherently scale-invariant.

### 3.2 Training and testing method

The RandomForestClassifier from sklearn. ensemble was used to ensemble multiple decision trees for Model 1 and Model 2, each splitting the data into two groups based on thresholds to reduce the Gini Index (National Institute of Food and Drug Safety Evaluation, 2019; Machado, Mendoza, and Corbellini, 2015). Random Forest was selected because it can handle nonlinear relationships and mixed feature types without requiring feature scaling. The dataset was divided into training and testing sets using a stratified 70:30 split to preserve class distributions. Each tree was constructed using random bootstrap samples from the training data (Out-of-Bag, OOB). Node splits were determined during tree growth by randomly selecting the split that minimized impurity from a random subset of candidate variables.

**FIGURE 1:**
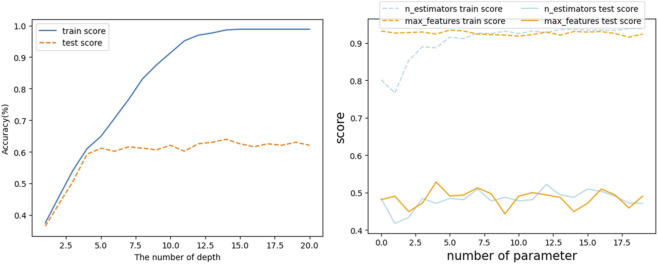
Hyperparameter Tuning: number of parameters (n_estimator, max_features, n_depth)^1^.

Instead of using GridSearchCV, which explores all hyperparameter combinations, the Bayesian Optimization from the bayes_opt library was used for hyperparameter tuning. This approach quantifies surrogate model uncertainty and identifies the next sampling location using an acquisition function (Frazier, 2018). In this study, the Bayesian Optimization (acquisition function) was employed to generate hyperparameter combinations (max_feature, max_samples, and n_depth) at each iteration. Bayesian Optimization was performed within predefined parameter ranges for tree depth, number of trees, and sampling proportions. Each hyperparameter combination was evaluated using stratified five-fold cross-validation, and the final model was selected based on the highest mean validation accuracy. Parameter search ranges were defined as follows: max_samples: 0.5–1.0, max_features: 0.5–1.0, n_estimators: 100–300, max_depth: 3–8 (Figure 1).

The performance of the Shortage Duration Prediction Model (Model 1) and six Shortage Occurrence Prediction Model (Model 2) was evaluated using classification evaluation metrics (precision, recall, and accuracy). Accuracy measures the proportion of correct predictions, precision reflects the proportion of actual positives among the predicted positive samples, and F1_score is the harmonic mean of precision and recall. These metrics were calculated using the macro-average, considering that both models are multiclass classifications (Chicco and Jurman, 2020; Caelen, 2017). Although duration classes were imbalanced, we reported macro-averaged precision, recall, and F1-scores to provide a balanced assessment across classes. The test set was kept fully isolated and was not used during model training or cross-validation to prevent information leakage. The training and testing data were generated using the train_test_split function, with the test_size set to 0.3.

$$accuracy=\frac{\left(True\;Positive\right)+\left(True\;Negative\right)}{\left(True\;Positive\right)+\left(True\;Negative\right)+\left(False\;Positive\right)+\left(False\;Negative\right)}$$

$$precision=\frac{\left(True\;Positive\right)}{\left(True\;Positive\right)+\left(False\;Positive\right)}$$

$$F1\_score=\frac{2\;\cdot \left(True\;Positive\right)}{2\cdot \left(True\;Positive\right)+\left(False\;Positive\right)+\left(False\;Negative\right)}=2\cdot \frac{precision\cdot recall\;}{precision+recall}$$

## 4 Results

Table 1 shows a summary of 1,054 cases of drug shortages reported by pharmaceutical companies to the KMFDS between 2018 and 2024. The data were categorized based on key attributes, including those related to drug shortage events, drug supply monitoring system, drug manufacturing, and drug characteristics.

**TABLE 1:** Summary of drug shortage cases in South Korea.

a. Related with drug shortage events	Count
Shortage incidence frequency (n = 888^2^)	
1 time	777
2 times	80
3 times	18
4 times	7
5 times	4
6 times	1
9 times	1
Shortage cause (n = 1116^3^)	
a. Increased demand	83
b. Decreased demand	6
c. Troubles in raw material supply	184
d. Regulatory issues	113
e. Supply chain management issues	272
f. Business decision	429
g. Others/Unknown	29
Shortage duration range (n = 1053^4^)	
0–30 days (<1 month)	100
31–180 days (1 month to <6 months)	196
181–360 days (6 months to <12 months)	34
361 days or more (≥12 months)	331
Suspension	392
Shortage incidence timing as of COVID pandemic declaration (n = 1054)	
Before-pandemic	669
After-pandemic	385

b. Related with drug supply monitoring system (n = 1054)	Count (Yes/No/Unknown)
Drugs required mandatory supply maintenance	(112/ 940/ 2)
Essential drugs designated by South Korea	(828/ 226/ 0)
Essential drugs designated by WHO	(731/ 323/ 0)
Drugs required supply discontinuation reporting	(244/ 810/ 0)

c. Related with drug manufacturing	Count
Imported/Domestic	
Imported products	523
Domestic products	463
Unknown	68
Type of manufacturing site	
Contract manufacturing organization (CMO)	761
In-house manufacture	293
Business size of the marketing authorized company	
Small (<100 billion KRW)	253
Medium (100 billion to <1 trillion KRW)	449
Large (≥1 trillion KRW)	82
Unknown	270

d. Related with drug characteristics	Count
Drug classification: OTC vs. ETC	
ETC	123
OTC	931
Drug classification: Type of ingredients	
Chemical	897
Bio	143
Oriental medicine	14
Single-agent drug vs. combination drug	
Single-agent drug	915
Combination drug	139
Routes of drug administration	
Oral	508
Injectable	385
Topical	153
Other	1
Unknown	7
Year of approval	
1960s–1970s	44
1980s–1990s	293
≥2000s	717
Existence of alternative drugs with same ingredients	
Yes	323
No	634
Unknown	972
National health insurance reimbursement	
Reimbursable	753
Non-reimbursable	257
Delisted^5^	44

### 4.1 Model 1 (Shortage duration range prediction model)

**FIGURE 2:**
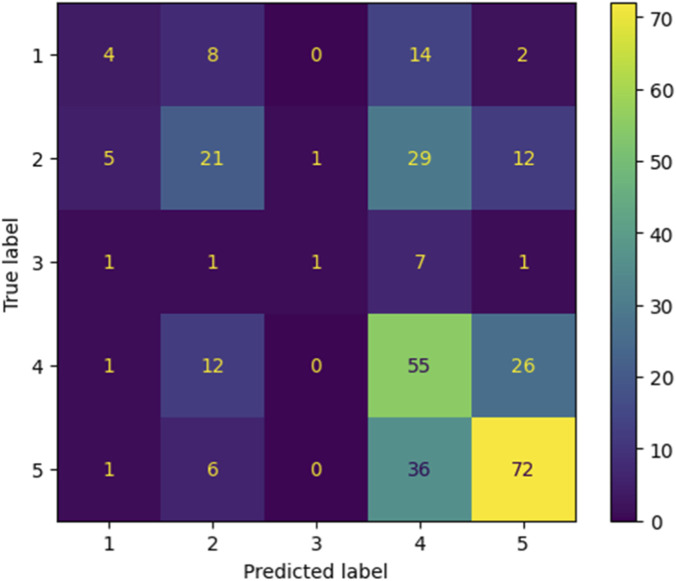
Confusion matrix per class (Model 1).

A baseline RandomForestClassifier using default hyperparameters achieved 0.43 accuracy under the same train–test split. After Bayesian Optimization, the optimized Model 1 reached 0.62 accuracy, demonstrating clear improvement over the baseline. Figure 2 and Table 2 shows that the optimized model reduced misclassification across several duration categories compared with the baseline, supporting the observed performance gain (Table 2).

**TABLE 2:** Classification report and confusion matrix of shortage duration range prediction model (Model 1).

Class (duration)	Precision	Recall	F1-score
0–30 days (<1 month)	1.00	0.03	0.05
31–180 days (1 month to <6 months)	0.54	0.67	0.60
181–360 days (6 months to <12 months)	1.00	0.17	0.29
361 days or more (≥12 months)	0.61	0.54	0.58
Suspension	0.66	0.88	0.75
Accuracy	​	​	0.62
Macro average	0.76	0.46	0.45

**FIGURE 3:**
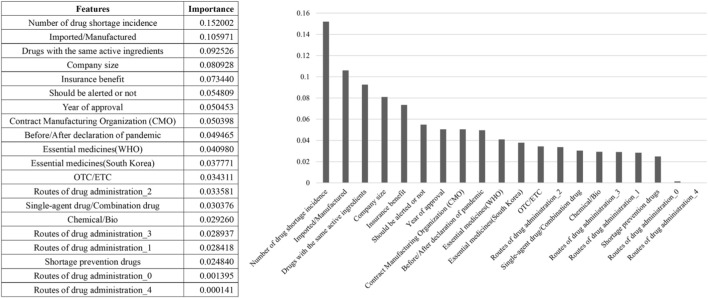
Feature importance of shortage duration range prediction model (model 1).

The three features with the highest importance for predicting the Shortage Duration Range were the Shortage Incidence frequency (0.152), Imported/Domestic (0.106), and Existence of alternative drugs with same ingredients (0.093). Business size of the Marketing Authorized Company (0.081) and National Health Insurance reimbursement (0.073) also showed high importance (Figure 3).

### 4.2 Model 2 (Shortage occurrence prediction model by each shortage cause)

Across all six one-vs-rest models predicting individual shortage causes, accuracy exceeded 0.70 after Bayesian Optimization, indicating stable performance across categories. Weighted precision, recall, and F1-scores also remained above 0.70, demonstrating that each model reliably identified its corresponding shortage cause (Table 3). Table 4 shows the feature importance values of the top five features in each model. Supplementary Table S2 shows the feature importance values for all features. Supplementary Figure S1 visualizes feature importances for all features.

**TABLE 3:** Classification report of shortage occurrence prediction model by each shortage cause (Model 2).

Shortage cause	Accuracy	Precision (macro/Weighted)	Recall (macro/Weighted)	F1-score (macro/Weighted)
Increased demand	0.93	0.97/0.94	0.53/0.93	0.55/0.91
Decreased demand	0.99	0.50/0.98	0.50/0.99	0.50/0.99
Troubles in raw material supply	0.80	0.90/0.84	0.51/0.80	0.47/0.71
Regulatory issues	0.95	0.47/0.90	0.50/0.95	0.49/0.92
Supply chain management issues	0.77	0.77/0.77	0.60/0.77	0.61/0.72
Business decision	0.74	0.73/0.74	0.73/0.74	0.73/0.74

**TABLE 4:** Top five feature importances of shortage occurrence prediction model by each shortage cause (Model 2).

Features	Feature importance
Cause a. Increased demand (due to the COVID-19 pandemic and other reasons)	
Shortage incidence frequency	0.159860
Year of approval	0.077193
Business size of the marketing authorized company	0.076793
Existence of alternative drugs with same ingredients	0.072283
Shortage incidence timing as of COVID pandemic declaration	0.069857
Cause b. Decreased demand (patent expiration, new competitors, and developments of substitutes)	
Existence of alternative drugs with same ingredients	0.212930
Essential drugs designated by WHO	0.140258
Drugs required supply discontinuation reporting	0.135990
Imported/Domestic	0.102378
Type of manufacturing site	0.062515
Cause c. Troubles in raw material supply (shortage, quality degradation, delivery delay, price increase, and contract expiration raw materials)	
Shortage incidence frequency	0.123844
Business size of the marketing authorized company	0.108332
Existence of alternative drugs with same ingredients	0.093989
Year of approval	0.071775
National health insurance reimbursement	0.063433
Cause d. Regulatory issues (license revocation, suspension of production/import/sales, recall/destruction, import/customs issues)	
Business size of the marketing authorized company	0.104231
Existence of alternative drugs with same ingredients	0.082905
Imported/Domestic	0.081812
Year of approval	0.074065
National health insurance reimbursement	0.072294
Cause e. Supply chain management issues (capacity and production line limitations, manufacturing site loss and change, facility obsolescence and failure, and product defects)	
Shortage incidence frequency	0.110314
Business size of the marketing authorized company	0.104795
Imported/Domestic	0.078509
Year of approval	0.076567
Existence of alternative drugs with same ingredients	0.072971
Cause f. Business decision (mergers and acquisitions, new product for substitution launches, low profitability, management difficulties, and deteriorating management)	
Business size of the marketing authorized company	0.115123
Shortage incidence frequency	0.096659
Existence of alternative drugs with same ingredients	0.077964
Imported/Domestic	0.076190
Year of approval	0.075517

The top five features with the highest importance in predicting shortages caused by “Shortage Cause a” (Increased demand) were Shortage Incidence frequency (0.160), Year of Approval (0.077), Business size of the Marketing Authorized Company (0.077), Existence of alternative drugs with same ingredients (0.0723), and Shortage incidence timing as of COVID pandemic declaration (0.070).

The top four features with the highest importance in predicting shortages caused by “Shortage Cause b” (Decreased demand) were Existence of alternative drugs with same ingredients (0.213), Essential drugs designated by WHO (0.140), Drugs required supply discontinuation reporting (0.136), and Imported/Domestic (0.102).

The top three features with the highest importance in predicting shortages caused by “Shortage Cause c” (Troubles in raw material supply) were Shortage Incidence frequency (0.124), Business size of the Marketing Authorized Company (0.108), and Existence of alternative drugs with same ingredients (0.094).

The most important feature in predicting shortage caused by “Shortage Cause d” (Regulatory issues) was Business size of the Marketing Authorized Company (0.104). Compared to the prediction models for other shortage causes, the feature importance values were relatively uniform. The difference in importance among features, excluding Business size of the Marketing Authorized Company (the most important), Routes of drug administration_0 and Routes of drug administration_4 (the least important), was <0.01.

The most important feature in predicting shortage caused by “Shortage Cause e” (Supply chain management issues) was Shortage Incidence frequency (0.110), followed by Business size of the Marketing Authorized Company (0.105).

The most important feature in predicting shortage caused by “Shortage Cause f” (Business decision) was Business size of the Marketing Authorized Company (0.115), followed by the Shortage Incidence frequency (0.097).

Taken together, the results show that Shortage incidence frequency and Business size of the Marketing Authorization Holder consistently ranked among the top predictors across several shortage causes, indicating their broad influence. In contrast, decreased-demand shortages showed high importance for substitutability-related variables, reflecting the different dynamics underlying this category. Overall, the feature-importance patterns suggest that each shortage cause is associated with a distinct set of determinants.

## 5 Discussion

The study found that the primary factor for predicting the shortage duration range in South Korea is the Shortage Incidence frequency. A repeated history of shortages reflects persistent supply-side vulnerabilities—such as limited manufacturing capacity, unstable access to APIs, insufficient redundancy across suppliers, or recurring quality-control issues—which remain unresolved over time and increase the likelihood that the same product will experience future disruptions.

The results indicated that the Shortage Incidence frequency, Business size of the Marketing Authorized Company, and Existence of alternative drugs with same ingredients were highly important in most shortage causes. These findings suggest that products marketed by larger Marketing Authorized Companies tend to have more stable production capability, whereas drugs that do not have therapeutically equivalent alternatives face greater risk during supply interruptions. Because these features are all supply-side characteristics, the results indicate that production and supply-chain constraints exert a stronger influence on drug shortages in South Korea than demand-side factors.

The study period (2018–2024) encompassed both pre-pandemic and post-pandemic environments, enabling evaluation of whether COVID-19–related supply-chain disruptions altered shortage determinants. Although national shortage counts fluctuated during the pandemic, the ranking of key predictors remained stable, indicating that the determinants identified in this study represent underlying structural characteristics rather than temporary pandemic-specific effects.

Drug shortages arise from a complex interplay of factors, encompassing supply and demand issues, regulatory challenges, and quality concerns, with various risks and frequencies depending on the drug characteristics (Mazer-Amirshahi et al., 2014). These factors vary depending on national healthcare systems, pharmaceutical market structures, and government regulations (Gray and Manasse, 2012; Acosta et al., 2019). The risk and occurrence patterns of drug shortages also differ according to manufacturer characteristics, domestic production versus importation status, therapeutic class, route of administration, and whether a drug is branded or a generic product (Fox et al., 2014; Giammona et al., 2020). Generic drugs, in particular, are more susceptible to shortages due to low profitability and limited production capacity, and this susceptibility is further exacerbated by quality-control issues and the fragile supply chain of APIs (Fox et al., 2014; United States Government Accountability Office, 2016).

The U.S. pharmaceutical market faces challenges (factors) that drive drug shortages, including limited incentives for producing low-margin medicines, inadequate evaluation and compensation for quality control in manufacturing, and logistical and regulatory challenges in ensuring a stable drug supply (U.S. Food and Drug Administration, 2020). In contrast, most drug supply disruptions in Canada arise from supply-related issues, including pharmaceutical quality control problems, production delays, recalls, regulatory actions, product suspensions, and the unavailability of raw materials (The Multi-Stakeholder Steering Committee on Drug Shortages, 2017). In China, drug supply disruptions are primarily attributed to unprofitable pricing. Price competition in the drug market and government-imposed price reduction policies, which lower prices to unprofitable levels, are identified as key contributors to drug shortages (Yang et al., 2016). Previous research also reports that middle-income countries experience additional challenges such as licensing delays and limited raw materials, while low-income countries face more severe shortages due to insufficient research capacity and weak policy frameworks (Gray and Manasse, 2012). These findings show that drug shortages in many countries are driven predominantly by supply-side factors, which is consistent with the pattern observed in South Korea.

In high-income nations, manufacturing issues, business decisions, raw material shortages, and regulatory challenges are the primary factors contributing to drug shortages. In middle-income countries, similar issues prevail, but additional factors, such as licensing delays and insufficient raw materials for local manufacturers, also play a significant role. In contrast, low-income countries face exacerbated shortages due to limited research, insufficient data, and weak policy frameworks (Gray and Manasse, 2012). These international patterns demonstrate that drug shortages are largely driven by supply-side vulnerabilities, which aligns with the situation in South Korea, where supply-side factors were also found to be highly influential in this study.

Research on drug shortages has been more extensive in developed nations than in South Korea, and many of the insights identified in these countries are relevant to the Korean context. Therefore, addressing drug shortages in South Korea may benefit from applying and adapting strategies that have been effective in other high-income settings.

Complex machine-learning models often show strong internal fit but are vulnerable to overfitting, particularly when class distributions are imbalanced or when the number of predictors is large relative to the sample size. The risk of overfitting is especially important, as specific patterns learned from past data could hinder early recognition of new or atypical shortage signals. To mitigate this risk, the study incorporated multiple safeguards. First, a stratified 70/30 train–test split was used to preserve class proportions, and the test set was kept entirely independent during model development to prevent information leakage. Second, Random Forest–specific techniques such as bootstrap aggregation and out-of-bag (OOB) validation helped assess model performance from quasi-independent samples. Third, Bayesian optimization was applied to tune key hyperparameters including tree depth, sample size, and feature sampling rate, thereby preventing overly complex trees and improving generalizability. Collectively, these techniques reduced the likelihood of overfitting and contributed to the robustness of the final prediction models.

Since drug shortages are closely related to both supply and demand factors, the feature analysis of the study would be enhanced by integrating data from the supply side (i.e., pharmaceutical companies) for modeling. In addition, when the distribution of categorical features is skewed toward “'0,” indicating “unknown,” using out-of-bag bootstrap sampling may lead to the construction of a random forest model where many trees exclude data other than “0” from their samples. Among the 1,054 products analyzed in this study, only seven (0.66%) had an “unknown” route-of-administration label, a level of missingness too small to meaningfully influence model performance. Therefore, the low feature importance of Routes of drug administration in the Shortage Occurrence Prediction Models is likely attributable to the inherently limited relevance of the route-of-administration variable to shortage mechanisms rather than distortions caused by missing data.

This study showed features with high importance for predicting shortage duration causes based on drug shortage data in South Korea. Identifying the importance of each factor for predicting shortage duration helps to identify the duration when a shortage occurs and facilitates the rapid normalization of the drug supply. Analyzing the key features with high importance for each shortage cause would help establish strategies to identify the root cause of future shortages. With additional integration of supply-chain and production data from manufacturers, the proposed models could be interfaced with South Korea’s national drug shortage monitoring system to support real-time risk identification and regulatory response. Such applications would contribute to healthcare system resilience by shifting shortage management from reactive to proactive approaches.

Conducting shortage pattern research using data from various organizations within the drug market, such as pharmaceutical companies, community pharmacies, and hospital pharmacies, along with publicly available data, may contribute to the stability of drug supply in South Korea. Future refinement and practical implementation of the models will require collaboration among government regulators, marketing authorization holders and manufacturers, wholesalers, hospital and community pharmacies, and academic researchers. Integrating data across these stakeholders will help establish a more coordinated national framework for drug shortage prevention and management.

## 6 Limitation

This study was conducted to analyze drug shortages in South Korea in depth but has some limitations. First, this study selected 18 features based on data accessibility and relevance, but potentially important factors such as changes in patient behavior (e.g., changes in purchasing patterns or drug compliance), global supply-chain disruptions, and policy changes were not included due to data collection constraints. These factors were difficult to measure consistently and were not available in standardized, detailed datasets. Second, this study utilized data from 2018 to 2024, and data collection within a limited period may not fully reflect long-term trends in drug shortages. Third, the prediction model developed in this study mainly focused on supply-side factors, so interactions with demand-side factors could not be fully considered. Fourth, the distributions of shortage duration and shortage-cause categories were uneven, which may have biased the prediction models toward majority classes. We acknowledge that the current model does not incorporate specific imbalance-handling techniques (e.g., class-weight adjustments or resampling), which may limit predictive performance for rare but meaningful shortage types. Future work should explore tailored imbalance mitigation strategies to improve minority-class sensitivity. Fifth, external validation using independent datasets—such as those from hospitals, wholesalers, or community pharmacies—was not performed, limiting the ability to assess model performance in real-world or cross-institutional settings. Sixth, because the model was trained solely on South Korean regulatory data, its generalizability to other health systems with different pharmaceutical structures may be limited. Lastly, the predictive model proposed in this study requires additional empirical studies to verify real-world applicability and effectiveness. These limitations are expected to be supplemented in follow-up studies, thereby enhancing the accuracy and practicality of drug shortage prediction models and contributing to the development of more effective policies.

## 7 Conclusion

This study developed two machine-learning models—one predicting shortage duration ranges and another predicting shortage occurrence by cause—and identified shortage incidence frequency as the most important predictor of future shortage duration. South Korea must implement an integrated data-sharing system among stakeholders to enable a swift response to future shortages and ensure their rapid resolution. Data should be shared transparently and consistently to complement the current incomplete reporting system. Drugs that have experienced several shortages should have their supply chain continuously monitored. Furthermore, the shortage prediction program in South Korea, which focuses on supply-side features such as economic viability, may help prevent shortages or reduce their duration and contribute to strengthening healthcare system resilience. Effective nationwide implementation will require collaboration among government regulators, marketing authorization holders, manufacturers, wholesalers, hospital and community pharmacies, and academic researchers.

## Data Availability

The raw data supporting the conclusions of this article will be made available by the authors, without undue reservation.

## Footnotes

1 The left graph illustrates how the stratified_k_fold score changes with variations in n_estimators and max_features in the untuned raw model. In contrast, the right graph depicts how the stratified_k_fold score changes with variations in n_depth in the raw mode.

2 The number of ‘drugs’ corresponding to each frequency rather than the number of ‘shortage cases’ was counted.

3 Among 1,054 cases of drug shortages reported in total, some shortage incidents have two or more causes.

4 Shortage duration range of one product could not be verified, so it was excluded.

5 Drugs that were formerly listed as reimbursable but have been delisted.
