# Supplementary material for: Drug shortage in South Korea: machine learning-based prediction models and analysis of duration and causal factors
Source: Front Pharmacol. 2026 Jan 9;16:1608843. doi: 10.3389/fphar.2025.1608843 (PMC12827063; doi:10.3389/fphar.2025.1608843)
Supplement: Supplementary file 1 [file Supplementaryfile1.docx]

***Supplementary Material***

**Supplementary Table A.** Features selected, detailed contents, and data sources

| **Features selected** | **Detailed contents** | **Data sources** |
| --- | --- | --- |
| **a. Related with Drug shortage event** | |  |
| 1. Shortage incidence frequency | Frequency of shortage reports for the same drug | As of shortage reporting date until 2022  <https://nedrug.mfds.go.kr/pbp/CCBAF11>  after 2023  <https://nedrug.mfds.go.kr/pbp/CCBAF10> |
| 1. Shortage causes | 1. Increased demand: Surging demand due to public health emergencies e.g. COVID-19 2. Decreased demand: Meager demand due to the end of product life cycle e.g. patent expiration or competitor entry 3. Troubles in raw material supply: Shortage, quality degradation, delivery delay, price increase, and contract expiration raw materials 4. Regulatory issues: License revocation, suspension of production/import/sales, recall/destruction, import/customs issues 5. Supply chain management issues: Capacity and production line limitations, manufacturing site loss and change, facility obsolescence and failure, and product defects 6. Business decision: Mergers and acquisitions, new product for substitution launches, low profitability, management difficulties, and deteriorating management |  |
| 3. Shortage duration | The period from the expected date of shortage to the expected date of supply normalization  Supply discontinued drugs are classified into indefinite supply shortages(shortage lasting ≥ 361 days) and supply disruptions(suspension) Based onthe Drug Safety system, drugs reported as approval withdrawn, expired, closure, or administration decision(cancelation) are classified into supply disruptions and others into indefinite supply shortage |  |
| 4. Shortage incidence timing as of COVID pandemic declaration | Classified into before and after the declaration of the pandemic as of March 11, 2020, the date of the WHO declaration of the COVID-19 pandemic |  |
| **b. Related with drug supply monitoring system** | |  |
| 5. Drugs required mandatory supply maintenance | Drugs that are essential for patient care but are avoided in production or importation due to lack of economic viability, requiring conservation of price(Service 2023; Liu et al. 2021) | <https://www.hira.or.kr/bbsDummy.do?pgmid=HIRAA030019000000&WT.gnb=%EB%AA%A9%EB%A1%9D%ED%91%9C> |
| 6. Essential drugs designated by South Korea | Drugs that are selected through consultation between the Minister of Health and Welfare, the Minister of Food and Drug Safety, and ~~the heads of~~ relevant central administrative agencies, which are essential for public health, such as disease control and radioactivity prevention, but are difficult to supply stably only through market function(Machado, Mendoza, and Corbellini 2015) | https://nedrug.mfds.go.kr/pbp/CCBAA01 |
| 7. Essential drugs designated by WHO | The essential drugs that the WHO (World Health Organization) has been updating every 2 years since 1977(Chicco and Jurman 2020) | [WHO Model List of Essential Medicines - 23rd list, 2023](https://www.who.int/publications/i/item/WHO-MHP-HPS-EML-2023.02) |
| 8. Drugs required supply discontinuation reporting | Drugs designated by KMFDS for mandatory reporting when production, importation, or supply is interrupted (No. 3 to No. 8)   - No. 3: drugs with two or less other drugs with the same active ingredients - No. 4: drugs whose market share is 50% or more among products containing the same active ingredients - No. 5: drugs containing the top 100 active ingredients in the previous year’s health insurance claims - No. 6: drugs of ingredients and formulations registered in essential drugs (South Korea) or essential drugs (WHO) and with three or fewer manufacturers or importers - No. 7: biopharmaceuticals with unstable supply of raw materials - No. 8: drugs for the treatment of severe diseases | Drug list that subjects to reporting of production, import, and supply discontinuation  <https://nedrug.mfds.go.kr/pbp/CCBAD01> |
| **c. Related with drug manufacturing** | |  |
| 9. Imported/Domestic | Classified into imported product and manufactured product in South Korea | <https://nedrug.mfds.go.kr/searchDrug> |
| 10. Type of Manufacturing Site | Classified into Contract Manufacturing Organization (CMO) which is a pharmaceutical production facility that produces drugs on a consignment basis at the request of a manufacturer and In-house manufacture |  |
| 11. Business size of the Marketing Authorized Company | Based on the 2022 financial statements, companies with sales less than 100 billion KRW are classified into “small,” those with sales between 100 billion KRW and 1 trillion KRW into “medium,” and those with sales more than 1 trillion KRW into “large.”  (companies whose sales are indeterminable are classified as “unknown”) | <https://dart.fss.or.kr/> |
| **d. Related with drug characteristics** | |  |
| 12. Drug Classification: OTC/ETC | OTC; general pharmaceuticals  ETC; prescription-based pharmaceuticals | <https://nedrug.mfds.go.kr/searchDrug> |
| 13. Drug Classification: Type of ingredients | Classified into chemical, bio, oriental medication |  |
| 14. Routes of drug administration | Classified into oral medication, injectable medication, topical medication, and other (drugs whose route of administration is indeterminable are classified into unknown) | <https://www.health.kr/> |
| 15. Year of approval | Classified into the 1960s–70s, the 1970s–80s, the 1980s–90s, and after the 2000s | <https://nedrug.mfds.go.kr/searchDrug> |
| 16. Existence of alternative drugs with same ingredients | Whether drugs with identical main ingredient codes, designated by the Health Insurance Review and Assessment Service, exist(Caelen 2017) | <https://www.health.kr/> |
| 17. National Health Insurance reimbursement | Classified into reimbursable, Non-reimbursable, and delisted | <https://www.hira.or.kr/ra/medi/getHistoryList.do?pgmid=HIRAA030035020000&WT.gnb=%EC%9D%98%EC%95%BD%ED%92%88%ED%86%B5%ED%95%A9%EC%A0%95%EB%B3%B4> |
| 18. Single-agent drug/Combination drug | classified into single-agent drug and combination drug | <https://www.health.kr/> |

**Supplementary Table B.** Feature importance of shortage occurrence prediction model by each shortage cause (Model 2)

| **Feature** | **Feature Importance** |
| --- | --- |
| **Cause a. Increased demand** (due to the COVID-19 pandemic and other reasons) | |
| Shortage Incidence frequency | 0.159860 |
| Year of approval | 0.077193 |
| Business size of the Marketing Authorized Company | 0.076793 |
| Existence of alternative drugs with same ingredients | 0.072283 |
| Shortage incidence timing as of COVID pandemic declaration | 0.069857 |
| Imported/Domestic | 0.066527 |
| Type of Manufacturing Site | 0.058443 |
| Essential drugs designated by South Korea | 0.052757 |
| Essential drugs designated by WHO | 0.051221 |
| National Health Insurance reimbursement | 0.048583 |
| Drugs required mandatory supply maintenance | 0.045482 |
| Routes of drug administration_1 (Oral) | 0.039785 |
| Drugs required supply discontinuation reporting | 0.038477 |
| OTC/ETC | 0.035228 |
| Single-agent drug/Combination drug | 0.028988 |
| Routes of drug administration_2 (Injectable) | 0.027727 |
| Type of ingredients | 0.026051 |
| Routes of drug administration_3 (Topical) | 0.019917 |
| Routes of drug administration_0 (Unknown) | 0.004827 |
| Routes of drug administration_4 (Other) | 0.000000 |
| **Cause b. Decreased demand** (patent expiration, new competitors, and developments of substitutes) | |
| Existence of alternative drugs with same ingredients | 0.212930 |
| Essential drugs designated by WHO | 0.140258 |
| Drugs required supply discontinuation reporting | 0.135990 |
| Imported/Domestic | 0.102378 |
| Type of Manufacturing Site | 0.062515 |
| Essential drugs designated by South Korea | 0.050525 |
| Year of approval | 0.049252 |
| Business size of the Marketing Authorized Company | 0.045923 |
| OTC/ETC | 0.041319 |
| Single-agent drug/Combination drug | 0.035004 |
| Type of ingredients | 0.028793 |
| National Health Insurance reimbursement | 0.025785 |
| Shortage incidence timing as of COVID pandemic declaration | 0.021551 |
| Routes of drug administration_1 (Oral) | 0.020788 |
| Shortage Incidence frequency | 0.011246 |
| Drugs required mandatory supply maintenance | 0.008588 |
| Routes of drug administration_2 (Injectable) | 0.004015 |
| Routes of drug administration_3 (Topical) | 0.003141 |
| Routes of drug administration_0 (Unknown) | 0.000000 |
| Routes of drug administration_4 (Other) | 0.000000 |
| **Cause c. Troubles in raw material supply** (shortage, quality degradation, delivery delay, price increase, and contract expiration raw materials) | |
| Shortage Incidence frequency | 0.123844 |
| Business size of the Marketing Authorized Company | 0.108332 |
| Existence of alternative drugs with same ingredients | 0.093989 |
| Year of approval | 0.071775 |
| National Health Insurance reimbursement | 0.063433 |
| Imported/Domestic | 0.063138 |
| Essential drugs designated by WHO | 0.057102 |
| Shortage incidence timing as of COVID pandemic declaration | 0.055070 |
| Type of Manufacturing Site | 0.045924 |
| Drugs required supply discontinuation reporting | 0.045848 |
| Essential drugs designated by South Korea | 0.042281 |
| Drugs required mandatory supply maintenance | 0.039881 |
| Routes of drug administration_1 (Oral) | 0.038518 |
| OTC/ETC | 0.033280 |
| Type of ingredients | 0.029974 |
| Routes of drug administration_2 (Injectable) | 0.029158 |
| Single-agent drug/Combination drug | 0.028015 |
| Routes of drug administration_3 (Topical) | 0.021103 |
| Routes of drug administration_0 (Unknown) | 0.009336 |
| Routes of drug administration_4 (Other) | 0.000000 |
| **Cause d. Regulatory issues** (license revocation, suspension of production/import/sales, recall/destruction, import/customs issues) | |
| Business size of the Marketing Authorized Company | 0.104231 |
| Existence of alternative drugs with same ingredients | 0.082905 |
| Imported/Domestic | 0.081812 |
| Year of approval | 0.074065 |
| National Health Insurance reimbursement | 0.072294 |
| Type of ingredients | 0.065405 |
| Drugs required supply discontinuation reporting | 0.064817 |
| Shortage incidence timing as of COVID pandemic declaration | 0.058786 |
| Type of Manufacturing Site | 0.055138 |
| Shortage Incidence frequency | 0.053909 |
| Essential drugs designated by WHO | 0.051140 |
| Single-agent drug/Combination drug | 0.042479 |
| Routes of drug administration_2 (Injectable) | 0.041455 |
| Routes of drug administration_1 (Oral) | 0.038997 |
| Essential drugs designated by South Korea | 0.036849 |
| OTC/ETC | 0.028980 |
| Drugs required mandatory supply maintenance | 0.026541 |
| Routes of drug administration_3 (Topical) | 0.018451 |
| Routes of drug administration_0 (Unknown) | 0.001101 |
| Routes of drug administration_4 (Other) | 0.000644 |
| **Cause e. Supply chain management issues** (Capacity and production line limitations, manufacturing site loss and change, facility obsolescence and failure, and product defects) | |
| Shortage Incidence frequency | 0.110314 |
| Business size of the Marketing Authorized Company | 0.104795 |
| Imported/Domestic | 0.078509 |
| Year of approval | 0.076567 |
| Existence of alternative drugs with same ingredients | 0.072971 |
| Shortage incidence timing as of COVID pandemic declaration | 0.063075 |
| National Health Insurance reimbursement | 0.062335 |
| Type of Manufacturing Site | 0.059323 |
| Essential drugs designated by WHO | 0.048392 |
| Essential drugs designated by South Korea | 0.045823 |
| Type of ingredients | 0.045161 |
| Drugs required supply discontinuation reporting | 0.044632 |
| Single-agent drug/Combination drug | 0.038870 |
| Routes of drug administration_2 (Injectable) | 0.034121 |
| OTC/ETC | 0.033770 |
| Routes of drug administration_1 (Oral) | 0.030366 |
| Drugs required mandatory supply maintenance | 0.028622 |
| Routes of drug administration_3 (Topical) | 0.022180 |
| Routes of drug administration_0 (Unknown) | 0.000145 |
| Routes of drug administration_4 (Other) | 0.000031 |
| **Cause f. Business decision** (Mergers and acquisitions, new product for substitution launches, low profitability, management difficulties, and deteriorating management) | |
| Business size of the Marketing Authorized Company | 0.115123 |
| Shortage Incidence frequency | 0.096659 |
| Existence of alternative drugs with same ingredients | 0.077964 |
| Imported/Domestic | 0.076190 |
| Year of approval | 0.075517 |
| National Health Insurance reimbursement | 0.067402 |
| Type of Manufacturing Site | 0.060308 |
| Essential drugs designated by WHO | 0.056859 |
| Drugs required supply discontinuation reporting | 0.046966 |
| Shortage incidence timing as of COVID pandemic declaration | 0.045403 |
| Essential drugs designated by South Korea | 0.042544 |
| Type of ingredients | 0.036722 |
| Single-agent drug/Combination drug | 0.036108 |
| Routes of drug administration_2 (Injectable) | 0.035589 |
| Routes of drug administration_1 (Oral) | 0.035269 |
| OTC/ETC | 0.033585 |
| Drugs required mandatory supply maintenance | 0.032778 |
| Routes of drug administration_3 (Topical) | 0.025924 |
| Routes of drug administration_0 (Unknown) | 0.003091 |
| Routes of drug administration_4 (Other) | 0.000000 |

**Supplementary Figure A.** Feature importance of shortage occurrence prediction model by each shortage cause (Model 2)

| **Cause a. Increased demand**  (due to the COVID-19 pandemic and other reasons) | **Cause b. Decreased demand**  (patent expiration, new competitors, and developments of substitutes) |
| --- | --- |
| **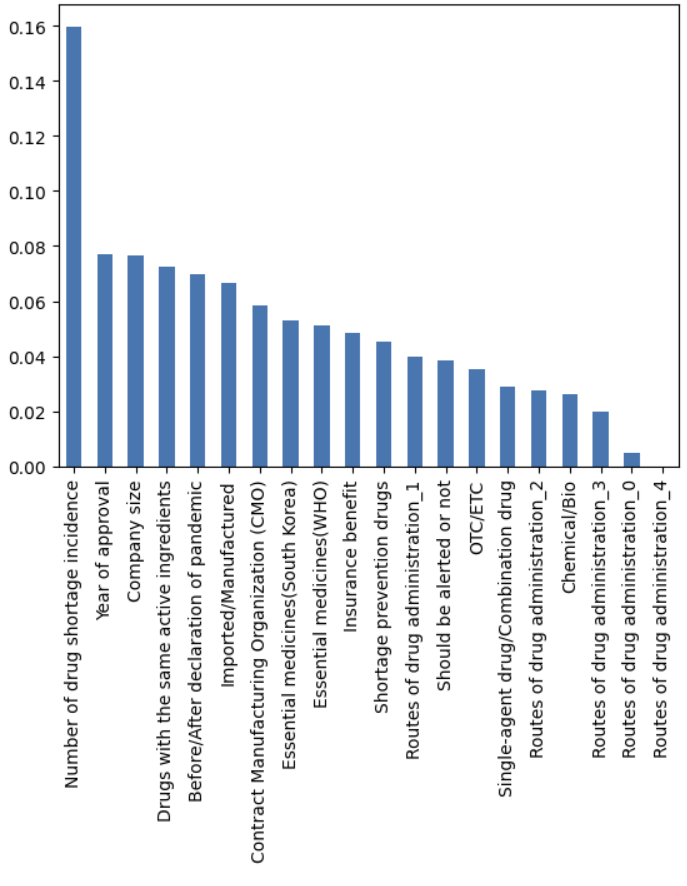** | **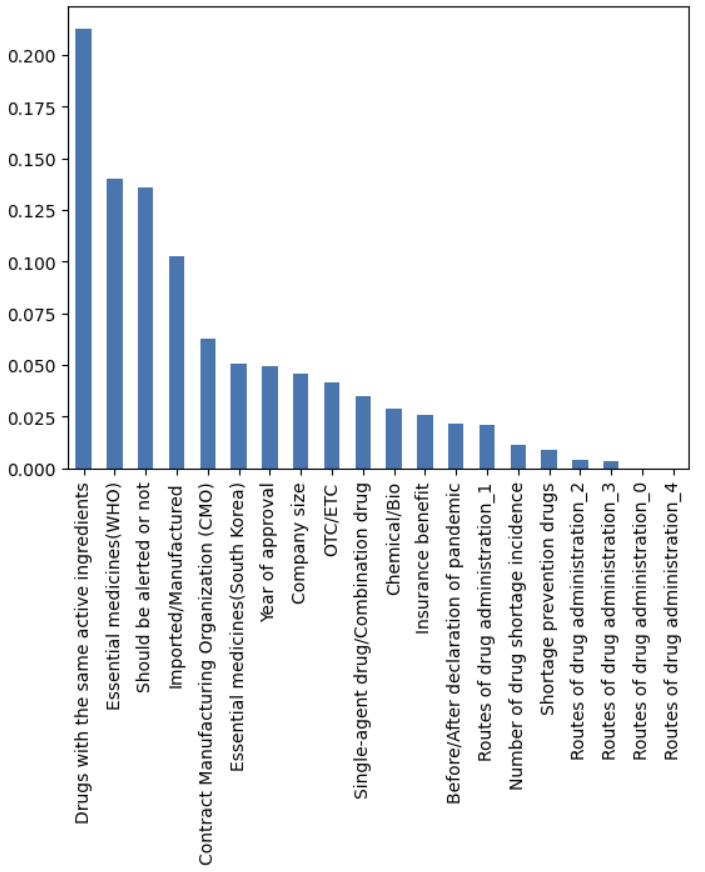** |
| **Cause c. Troubles in raw material supply**  (shortage, quality degradation, delivery delay, price increase, and contract expiration raw materials) | **Cause d. Regulatory issues**  (license revocation, suspension of production/import/sales, recall/destruction, import/customs issues) |
| **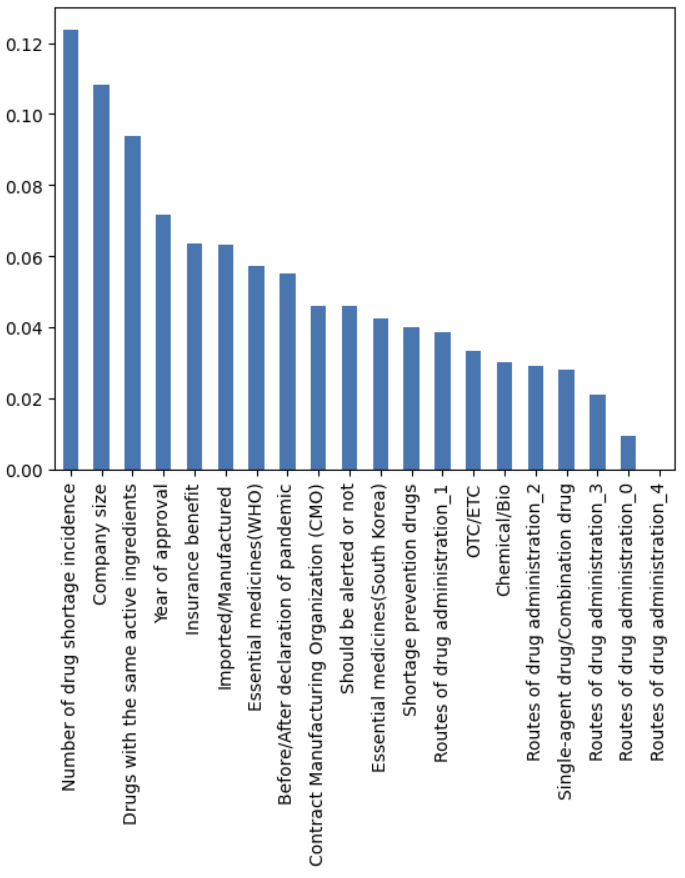** | **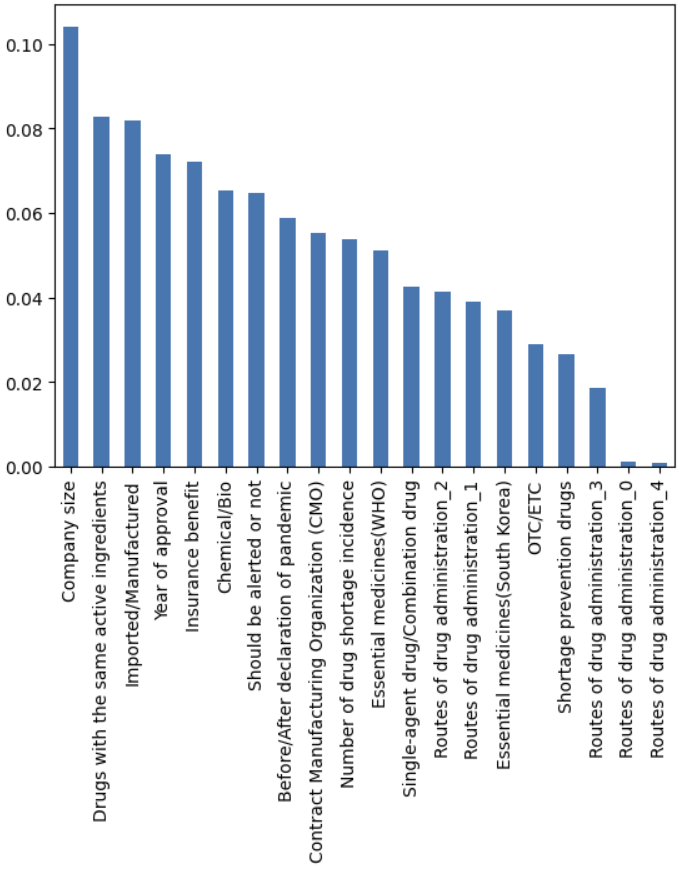** |
| **Cause e. Supply chain management issues**  (Capacity and production line limitations, manufacturing site loss and change, facility obsolescence and failure, and product defects) | **Cause f. Business decision**  (Mergers and acquisitions, new product for substitution launches, low profitability, management difficulties, and deteriorating management) |
| 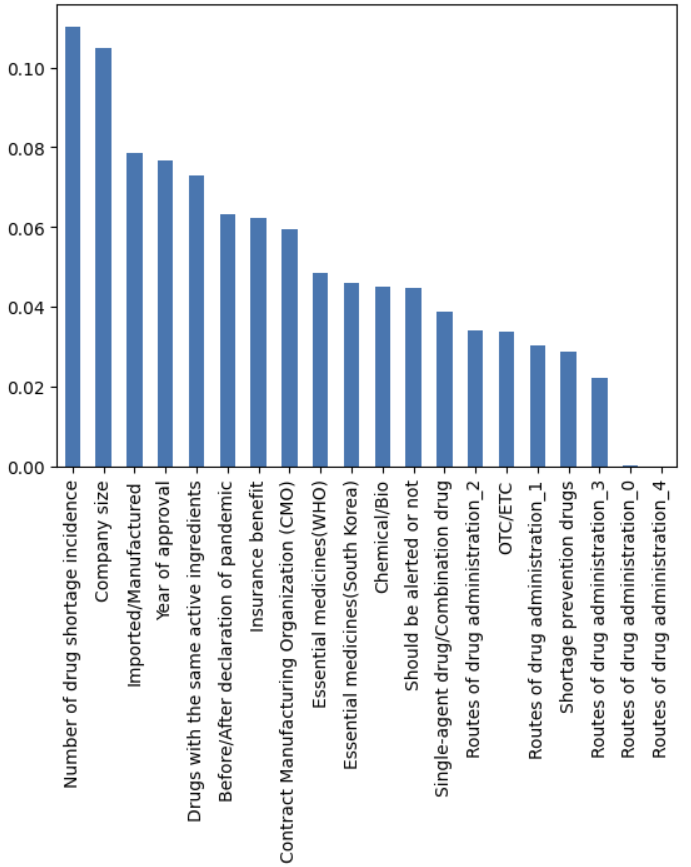 | 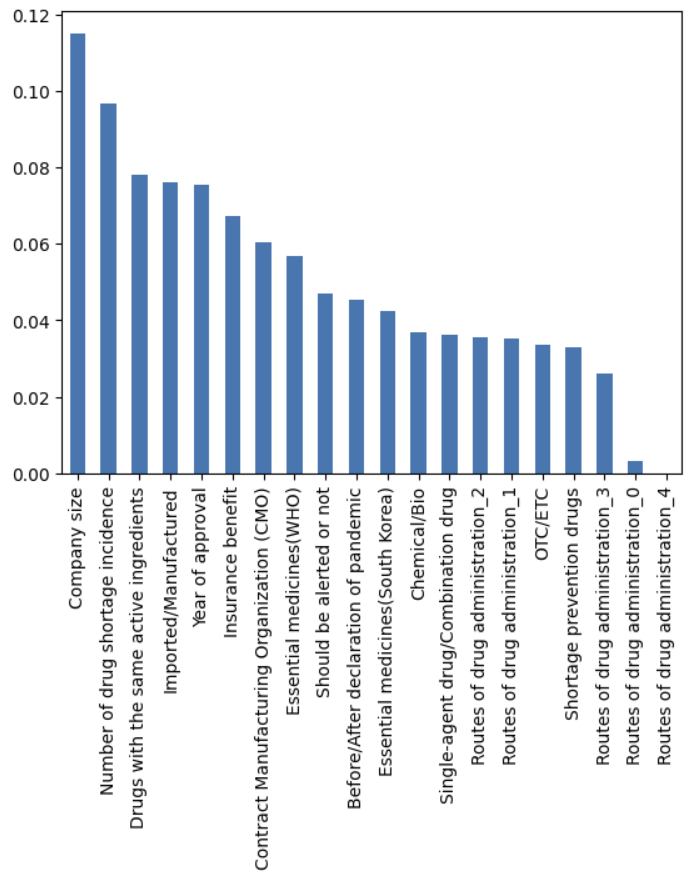 |
